# Development and validation of the Nursing Students’ Growth Mindset Scale (NSGMS)

**DOI:** 10.3389/fmed.2025.1550214

**Published:** 2025-11-05

**Authors:** Meijuan Cao, Zheyi Ling, Chunqi Xie, Xiaojuan Xu

**Affiliations:** ^1^School of Medicine and Nursing, Huzhou University, Huzhou, China; ^2^School of Nursing, Hangzhou Normal University, Hangzhou, China

**Keywords:** growth mindset, nursing students, scale development, nurse, scale

## Abstract

**Background:**

Growth mindset is increasingly valued for its important role in the training of nursing students. However, research on the development of tools to measure growth mindset effectively among nursing students is limited.

**Objective:**

To develop a measurement tool to evaluate nursing students’ growth mindset.

**Design:**

A cross-sectional methodological study.

**Participants and setting:**

A total of 271 nursing students from 4 universities or university colleges in Zhejiang and Hunan participated in the study.

**Methods:**

Domains and items of nursing students’ growth mindset were identified and created by conducting a literature review and qualitative interviews. To test the content validity, a 2-round Delphi was adopted, and a pilot implementation was conducted with 30 nursing students. The construct validity of the scale was tested using exploratory and confirmatory factor analyses (*n* = 206).

**Results:**

According to the exploratory factor analysis results, the Kaiser–Meyer–Olkin value was 0.87, and the Bartlett test’s χ^2^ was 2659.680 (*p* < 0.05). One item with a factor loading value below 0.50 was removed from the scale, and the obtained factor structure explained 67.01% of the variance. The fit indices of the scale model tested in the confirmatory factor analysis were determined as χ^2^/df = 1.42, RMSEA = 0.05, CFI = 0.97, TFI = 0.97, IFI = 0.97. The scale structure was confirmed using 4 factors and 21 items. The Cronbach’s alpha values were found to be 0.77–0.90 for the subdimensions of the scale and 0.88 for the total scale. The data also showed good test–retest stability (ICC = 0.90).

**Conclusion:**

This scale is a reliable and valid measurement tool to determine nursing students’ level of growth mindset. Further studies with larger and more diverse samples are recommended to evaluate the psychometric structure of this scale.

## 1 Introduction

In the context of high-quality health services and nursing development driven by emerging technologies, the training of excellent nursing professionals faces new requirements and challenges. However, the development of nursing students lacks promise. Evidence (1) suggests that nursing students generally deal with academic anxiety and burnout and their scores are not always satisfactory. Meanwhile, nursing students’ psychological problems seem to be prominent, with medium-to-high levels of psychological stress (2, 3). Across the 21st century, the cultivation of mindset ability has become the core and primary goal of the educational reform of nursing students (4, 49). Different thinking patterns lead to obvious differences in students’ learning styles, academic achievement, and emotion regulation strategies (5). Nursing students are a powerful reserve force for professional nursing teams; therefore, the cultivation and development of their thinking modes are highly important.

Growth mindset as a multidimensional concept, interpreted differently by various scholars. In 2006, Dweck (6) first proposed the growth mindset theory. Growth mindset is defined as individuals’ beliefs that their efforts play a decisive role but without denying the role of talent. It was explained using five dimensions: encountering challenges, encountering obstacles, views on efforts, views on evaluation, and the success of others. On the basis of Dweck’s general definition of growth mindset Cooley and Larson (7) described growth mindset as comprising three basic areas: attitudes toward studying, response to feedback, and response to setback. Accordingly, Chinese researchers generally interpret growth mindset across seven dimensions (8, 9): intelligence, effort, self-cognition, facing setbacks, facing challenges, facing the evaluation of others, and facing the success of others. Based on these, the researchers provided a framework for developing a Growth Mindset Scale for nursing students.

An increasing number of researches have suggested that a growth mindset can be used to predict students’ academic success in their present and future professional development and has a significant positive impact on students’ personal ability, attitude, and mental health (10–12). For instance, students with a growth mindset are more willing to accept challenging assignments (13), continue to pursue new goals despite setbacks (14), and have better communication and people skills (15). In addition, a study on engineering students found that a growth mindset can foster their enterprising attitudes toward studying and healthy living habits (16). Cooley and Larson (7) found that a growth mindset positively affects the resilience, coping capital, and stereotypes of pharmacy educators and students. A cross-sectional study on 130 fourth-year veterinary students indicated that those with a growth mindset felt less anxious about work–life balance and future work (17). The development and use of an appropriate scale are indispensable for assessing the required level of growth mindset for nursing students. This can encourage further studies to look for evidence supporting a reasonable and efficient growth mindset.

Currently, most research instruments used to measure nursing students’ growth mindset level are universal (18), such as the Implicit Theories of Intelligence Scale-3 (ITIS-3; 20), Implicit Theories of Intelligence Scale-6 (ITIS-6; 21), and Growth Mindset Scale (19). In addition, many instruments developed for other health-related disciplines primarily focus on the acquisition of academic knowledge and the development of clinical reasoning, while paying less attention to nursing-specific situational challenges, such as procedural errors, communication conflicts, questioning by clinical supervisors, or patient refusal (20). These challenges can substantially influence nursing students’ beliefs about learning, effort, and coping with setbacks. Although the universal scales can be used as a reference, the pertinence and comprehensiveness of the measurement of nursing students’ growth mindset are restricted because of unique professional and workplace requirements. With the development and extension of healthcare, nursing students are expected to constantly acquire new knowledge goals and skills goals (21). This is consistent with the concept of a growth mindset, but no related items exist in the existing scales. The nursing profession serves individual throughout their lives and has a low tolerance rate for faults in theoretical studying and clinical practice. Nursing students are universally subjected to more stress when they fail (3). Growth-oriented thinking plays a crucial role in individual performance in the face of frustration and stress; therefore, it is necessary to consider adding the relevant items to scales. Moreover, nursing students have their own special professional core competencies, such as interpersonal communication skills (22). However, the relevant scale items remain unavailable to nursing students. SuTing (9) offered items for musical talent, such as “Anyone can gain musical talent by learning.” Furthermore, almost all specific scales focus on assessing primary and secondary school students’ growth mindset in the basic curriculum (23), but a specific scale for nursing students is not available.

Developing a growth mindset in nursing students is an essential issue, as it plays a key role in fostering competent nursing professionals across different levels. This study is grounded in the growth mindset model (24) and, combining with the characteristics of the nursing students, aims to develop a comprehensive growth-mindset assessment tool tailored to nursing students. The study first constructed an initial version of the scale through a systematic literature review and semi-structured interviews. After two rounds of Delphi expert consultations and subsequent revisions, a pilot test was conducted to assess feasibility. Finally, exploratory and confirmatory factor analyses, together with internal consistency and test–retest reliability assessments, were performed on a large sample to confirm the scale’s psychometric properties. This will provide a reliable basis for the corresponding promotion strategy and play a significant role in improving nursing students’ comprehensive abilities to promote high-quality nursing talent.

## 2 Population

### 2.1 Sampling

The inclusion criteria comprised (a) full-time nursing students who (b) consented to participate in the research. Exclusion criteria were as follows: (a) master’s or doctoral nursing students, (b) individuals who were not at school during the survey period, and (c) those who withdrew from the study. According to factor analysis requirements, the sample size should be at least 5–10 times the number of items (25), with an additional 20% allowance for non-responses, estimating a required sample size of 144–288 participants. In total,220 questionnaires were distributed (effective response rate: 93.6%), of which 206 questionnaires were valid.

In the study, 220 nursing students were recruited voluntarily. After excluding invalid data, 206 students were included in the analysis. Most students were 18 or 19 years old. Of these, 21 were male (10.2%) and 185 were female (89.8%). Among the participants, 113 students (54.8%) were in associate degree programs and 93 (45.2%) were in undergraduate programs.

### 2.2 Data collection

The convenience sampling method was used to recruit participants from four junior and undergraduate nursing colleges in the Zhejiang and Hunan provinces between September and December 2023. The researchers actively contacted the participating schools and obtained informed consent. The survey was administered both online and offline. Online surveys were conducted via email and online questionnaire platforms, whereas offline surveys were distributed in classes and dormitories. To ensure the quality of data collection, the researchers received uniform training and were familiar with the meaning of the questionnaire entries. Participants were recruited according to inclusion and exclusion criteria to ensure that they accurately reflected the target population’s characteristics and participated voluntarily. And the participants were informed about the research objectives, procedures, and other matters that needed attention; their anonymity was especially emphasized. The surveyors checked and corrected the completed questionnaires on-site. During data processing, problematic questionnaires were excluded, and the data were double-entered into Excel by two individuals, followed by logical verification before analysis.

## 3 Methods

### 3.1 Research design

This study was primarily framed by growth-mindset theory and analyzed nursing students’ growth mindset across the following key points: intelligence, effort, self-cognition, facing setbacks, facing challenges, facing the evaluation of others, and facing the success of others, thereby exploring the underlying framework for a nursing-student growth-mindset scale. In developing candidate items we referred to Dweck’s Implicit Theories of Intelligence Scales (3-item and 6-item versions) and a 20-item Growth Mindset Scale (26).

**Figure 1:**
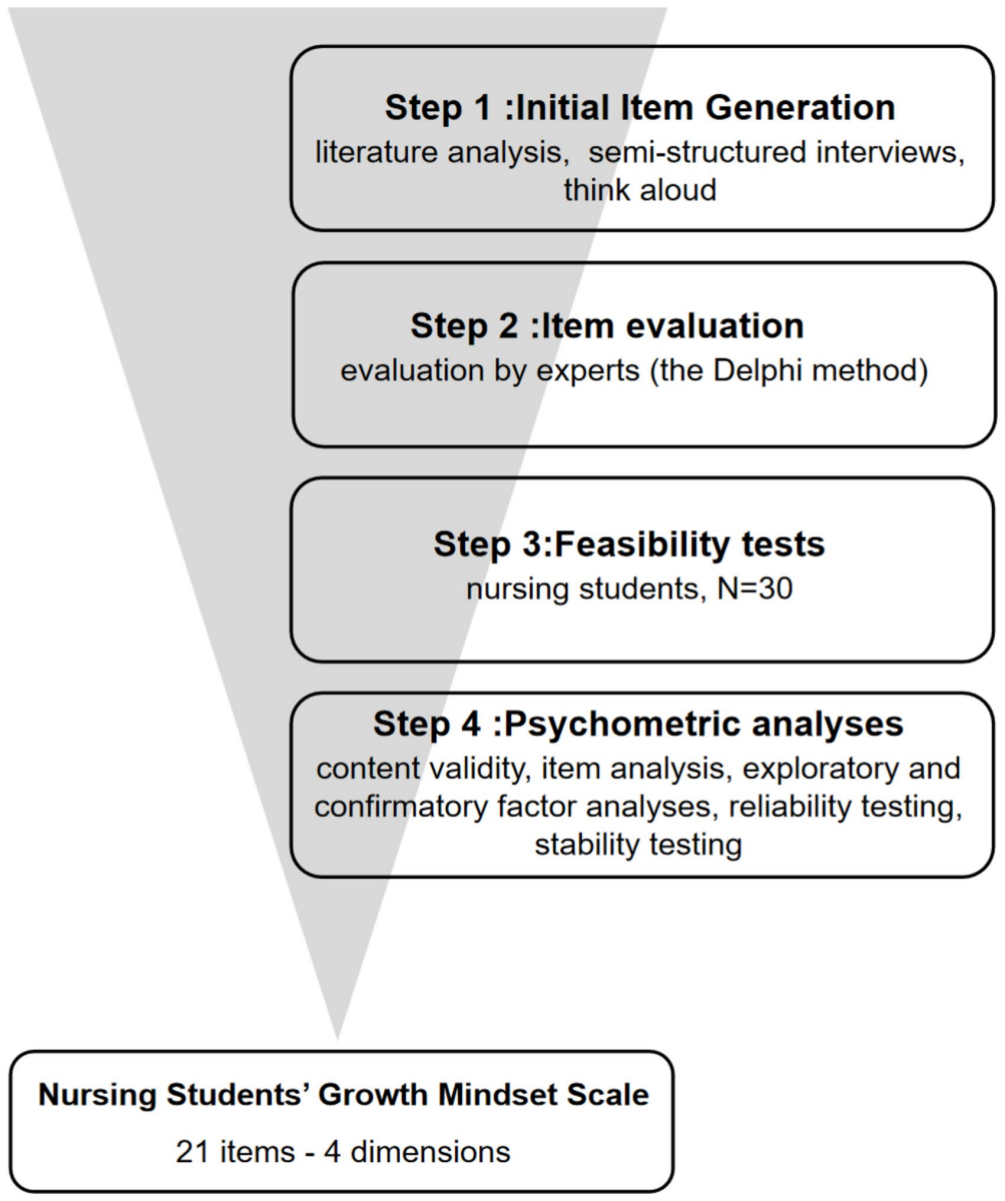
Development and validation of the nursing students’ Growth Mindset Scale in four steps.

The study employed a methodological approach combining literature analysis, qualitative interviews, the Delphi method, feasibility testing, and questionnaire surveys, and it aimed to develop and validate a growth-mindset scale tailored to nursing students. This study builds upon a well-regarded approach for developing scales in the field of nursing to ensure the reliability and validity of the scale (27, 28) (Figure 1).

### 3.2 Initial item generation

An initial version of the scale was formulated by generating an item pool grounded on growth mindset theory. To supplement and verify the nursing students’ characteristics, semi-structured interviews were conducted with 20 nursing students. Participants were freely recruited through the school and encouraged to express their views on intelligence and ability. They were asked about how they responded to challenges and setbacks in their professional studies and their attitudes toward others’ evaluations. Based on the principles of item formulation and repeated discussions, a draft Growth Mindset Scale for nursing students was developed, which included five dimensions: self-awareness and development, attitude toward challenges, attitude toward setbacks, attitude toward others’ success, and attitude toward others’ evaluations. It comprised a total of 24 items. A 5-point Likert scale was chosen, being the most widely used scale for measuring specific growth mindsets (9). The scale is precise because of its option to represent neutrality (29). The response choices provided included: “Strongly Disagree,” “Disagree,” “Neutral,” “Agree,” and “Strongly Agree.”

### 3.3 Delphi method

#### 3.3.1 Selection of experts

The inclusion criteria for experts were as follows: working in the fields of nursing education and psychological nursing at least 5 years; having at least a master’s degree or an associate-senior professional title in the field; and being willing to participate actively in the study. A total of 15 experts in the fields of nursing education and psychological nursing were invited to participate in the consultations.

#### 3.3.2 Development of expert consultation questionnaire

Two rounds of expert consultations, were conducted to evaluate the content validity of the initial scale. In each round, the experts were asked to complete a questionnaire, with items rated for importance using a 5-point Likert scale (1 = “Not Important,” 5 = “Very Important”) and relevance using a 4-point Likert scale (1 = “Not Relevant,” 5 = “Highly Relevant”). Each item was accompanied by a suggestion box with open-ended questions for each dimension to enable experts to provide advice. The entries were revised accordingly, with items removed if their mean importance score < 3.50 or their coefficient of variation > 0.25 (30).

#### 3.3.3 Distribution and collection of questionnaires

The questionnaires were distributed and collected by the research team members via email. Experts were reminded by telephone or SMS to return the consultation results. After the first round of consultation, the research team summarized and analyzed the expert opinions and formed a more complete questionnaire for the second round of consultation. After the second round, the questionnaire items were further revised and improved until the experts’ opinions reached a relatively consistent level.

### 3.4 Feasibility testing

The initial scale was pilot-tested with 30 nursing students. The completion time for each questionnaire was approximately 15–20 min. Cronbach’s alpha values for each dimension were ≥ 0.70, indicating acceptable internal consistency of the initial scale (31). However, some nursing students had difficulty understanding the term “peers” in the items. After a discussion, the term was revised to “classmates.”

### 3.5 Validation of scale

#### 3.5.1 Content validity

Item-level content validity index (I-CVI) and scale-level content validity index (S-CVI) were used to reflect the content validity. When the I-CVI is ≥ 0.78 and S-CVI is ≥ 0.8, the scale’s content validity is considered good (32).

#### 3.5.2 Item analysis

The critical ratio (CR; extreme value test) and correlation coefficient methods were utilized for item evaluation and selection. The total scores of the 206 questionnaires were sorted from high to low; the top 27% and the bottom 27%were designated as the high score group and the low score group, respectively. Independent samples t-tests (95% confidence interval) were performed to compare the differences between the two groups for each item, and items with a CR < 3.0 or *p* > 0.05 were removed (33). Subsequently, item-total score correlations were determined to evaluate item discrimination. An item was considered for removal if the correlation coefficient was < 0.40 (34).

#### 3.5.3 Construct validity

To determine construct validity, exploratory factor analysis (EFA) and confirmatory factor analysis (CFA) were applied. A Kaiser–Meyer–Olkin (KMO) test value > 0.6 and the significance level of Bartlett’ s test of sphericity (< 0.001) indicated that factor analysis could be performed. A principal component analysis and Kaiser-normalized varimax rotation were applied to evaluate the factor structure and several factors, and an eigenvalue > 1.00 was taken, combined with a gravel map (35). The study followed the criterion that the percentage of total variance explained by the data needs to range from 50 to 75% (36). Furthermore, items with factor loadings > 0.5 were acceptable (37). And then, the fit of the scale structure created in the EFA was determined using the CFA. The fit index criteria were χ^2^/df < 3, Root-Mean-Square Error of Approximation (RMSEA) < 0.08, Incremental Fit Index(IFI) < 0.9, Comparative Fit Index(CFI) > 0.9.

#### 3.5.4 Criterion-related validity

Criterion-related validity can be tested by identifying a previously validated instrument for measuring a similar concept (38, 39). The ITIS-6 was selected as the criterion because it has been used widely to assess students’ growth mindset levels. It comprises 6 items and uses a 6-point Likert scale. The score ranges from 6 to 36, the higher the better. The Cronbach’s alpha coefficient of ITIS-6 was confirmed as 0.78 (40). Correlation coefficients ≥ 0.5 (*p* < 0.05) indicated good criterion-related validity (38).

#### 3.5.5 Reliability testing

The internal consistency of the scale was evaluated by measuring Cronbach’s alpha. A Cronbach’s alpha value > 0.7 is considered to indicate acceptable reliability (41).

#### 3.5.6 Stability testing

To determine the stability of the scale, its retest reliability was measured. The tests were conducted twice for each group of respondents using the same questionnaire. Pearson’s correlation coefficients ≥ 0.7 are considered to indicate an acceptable level of stability (42). It has been suggested that a retest should occur after an interval of 2–4 weeks with a sample size of 20–30 participants (43). In this study, 30 nursing students were randomly selected from the sample for a retest two weeks later.

### 3.6 Data analysis

SPSS version 26.0 was utilized for data analysis, and the general demographic data of the students was analyzed by descriptive statistics, and the statistical significance was set at *p* < 0.05. The CR and correlation coefficient methods were utilized to evaluate and select the items. An EFA was performed to extract the common factors and classify the items. Following the EFA, a CFA was applied to evaluate the scale structure (35, 44). The internal consistency of the scale was confirmed via the Cronbach’s alpha coefficient (41). In addition, the stability of the scale was assessed via test–retest reliability (42).

### 3.7 Ethical considerations

This study was approved by the Ethics Committee of the Hangzhou Normal University (approval number: 2023060). Written or spoken informed consent was obtained from each participant, and the purpose and processes of the study were explained to all participants. During the investigation, the investigator explained that the participants have right to refuse or withdraw at any time and that their privacy would be strictly protected. The data were stored in a locked data cabinet and used solely for academic research.

## 4 Results

### 4.1 Content validity

On the basis of expert recommendations and discussions among the research group, the pre-test version was finalized with 24 items in 5 dimensions. Seventeen items from the original item pool were revised for language and expression. One item with hybrid content was divided into two, two items were combined, two were deleted, and two new items were added. The order of items was also adjusted according to the dimensions. The deleted items were as follows: “I will continue to learn to improve my nursing professional skills” and “The nursing profession relies on rote memorization and does not require intensive study.” In this study, the authority coefficients (Cr) of the expert panels in the two rounds of Delphi consultation were both ≥ 0.8, indicating a high level of expert authority. The I-CVI in the first round ranged from 0.8 to 1.0, and the S-CVI was 0.958. In the second round, the I-CVI was 0.833–1.0, and the S-CVI was 0.923, showing satisfactory content validity of the scale (32).

### 4.2 Item analysis results

The CR analysis revealed that all items had a CR value > 3 and significant differences in item scores between two groups (*p* < 0.05; 35). Pearson’s correlation coefficient analysis showed that the item-total score correlations of two items were < 0.40, leading to their removal (34), as detailed in Table 1. The entries deleted from the scale were “My classmates are better than me, and I feel like a failure” and “The excellence of my classmates has nothing to do with me, and I will not change for it.”

**Table 1:** Item analysis results.

Item no.	CR	*r*
1	11.369***	0.581
2	16.128***	0.670
3	12.518***	0.575
4	10.631***	0.545
5	12.717***	0.587
6	8.913***	0.527
7	12.092***	0.592
8	12.812***	0.568
9	12.790***	0.631
10	12.571***	0.579
11	8.819***	0.457
12	11.05***	0.582
13	11.061***	0.528
14	8.17***	0.465
15	10.6***	0.540
16	9.444***	0.418
17	4.505***	0.381
18	6.818***	0.387
19	7.436***	0.426
20	6.471***	0.497
21	8.123***	0.417
22	8.052***	0.452
23	8.157***	0.559
24	8.994***	0.501

***p < 0.001.

### 4.3 Exploratory factor analysis

Exploratory factor analysis (EFA) of the retained items produced a KMO value of 0.87, while Bartlett’s test of sphericity returned a chi-square of 2659.680 (*p* < 0.05), confirming the appropriateness of factor analysis. Principal Component Analysis with Varimax rotation identified four factors with eigenvalues ≥ 1.00, explaining a cumulative variance of 67.015%. The factor loadings after the rotation are listed in Table 2. Based on the EFA results, the item “Teachers/patients/classmates criticize me; I will be afraid to contact them again,” with a factor loading < 0.5, was removed (37). The analysis also revealed that items related to “attitude toward others’ success” and “attitude toward others’ evaluations” clustered into a single dimension. “I believe that with my learning abilities, I can achieve certain success in the field of nursing” was merged into the “attitude toward challenges” dimension. After discussions, to better reflect the characteristics and practical significance of nursing students’ growth mindset, the dimension “self-recognition and development” was renamed “perception of talent and intelligence,” and “attitude toward others’ success” and “attitude toward others’ evaluations” were combined and renamed “attitude toward others’ influences.” After the analysis, the final scale included four dimensions: “perception of talent and intelligence,” “attitude toward challenges,” “attitude toward setbacks,” and “attitude toward others’ influences,” comprising a total of 21 items (Table 2).

**Table 2:** Factor loadings of items in the draft scale.

No.	Items	Factors*			
		F1	F2	F3	F4
1	I believe intelligence is innate and cannot be changed.		0.782		
2	I believe some students are naturally more suited to studying nursing.		0.841		
3	My abilities are limited, and I cannot achieve success in the nursing profession.		0.865		
4	I believe intelligence can be improved through effort.		0.835		
5	Effort is more important than talent in studying nursing.		0.873		
6	I believe that with my learning abilities, I can achieve certain success in the field of nursing.	0.813			
7	I am willing to participate in activities related to the profession, such as nursing skills competitions and entrepreneurship competitions.	0.806			
8	I can actively face the challenges of life, illness, and death that come with the nursing profession.	0.852			
9	I can actively face various challenges in learning nursing theory courses, such as dealing with the large amount of content to memorize.	0.874			
10	I am willing to explore new challenges in the process of learning nursing skills.	0.888			
11	I am not afraid to face various challenges during clinical observations and internships.	0.779			
12	Not being able to answer the questions from teachers or patients makes me feel frustrated.			0.856	
13	When my grades in nursing courses are unsatisfactory, I feel that I am not suited for the nursing profession.			0.802	
14	I dare to face the shortcomings I am exposed to during the nursing study process.			0.790	
15	When my grades in nursing courses are unsatisfactory, I will analyze the reasons for my failure in order to solve the problem.			0.815	
16	When encountering difficulties in the nursing study process, I will actively seek help from others.			0.813	
17	When classmates are better than me, I believe it is because of their hard work.				0.720
18	When classmates are better than me, I will actively learn from their successful methods.				0.795
19	When teachers, patients, or classmates praise me, it is because I am well-suited to studying nursing.				0.660
20	Criticism from teachers, patients, or classmates helps me improve my nursing abilities.				0.772
21	When teachers, patients, or classmates praise me, it is because I have put in a lot of effort in my nursing studies.				0.841

*F1, attitude toward challenges; F2, perception of talent and intelligence; F3, attitude toward setbacks; F4, attitude toward others ‘influences.

### 4.4 Confirmatory factor analysis

**Figure 2:**
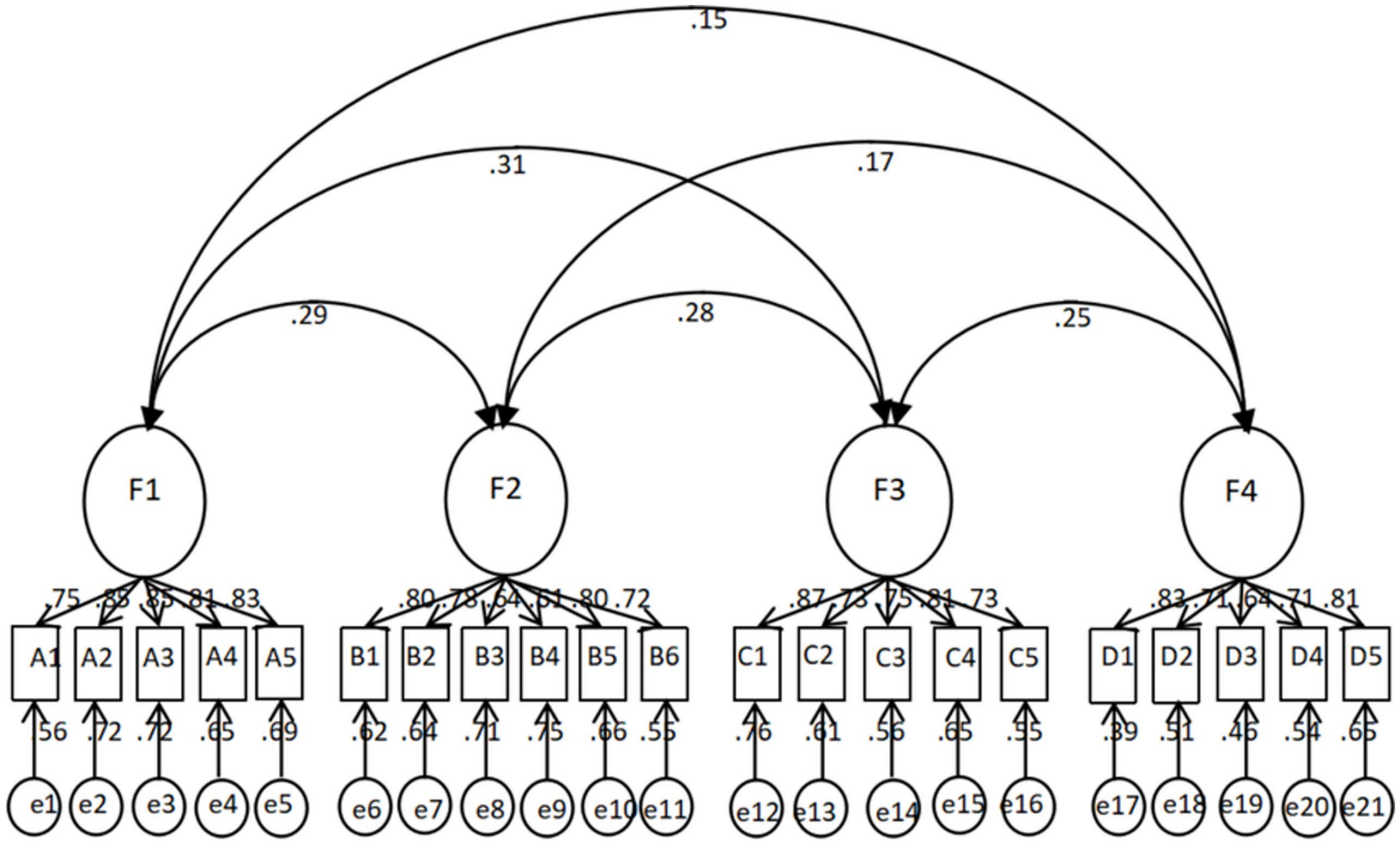
CFA results for the nursing students’ Growth Mindset Scale.

The CFA was applied to test the construct validity of the NSGMS, and the fit indices of the tested model were determined as χ^2^/df = 1.42, RMSEA = 0.045, IFI = 0.970, CFI = 0.969, PGFI = 0.710, PNFI = 0.788, PCFI = 0.845. All model fit indices reached the standard (44), as shown in Table 3. The structural model is illustrated in Figure 2.

**Table 3:** Fit index values of the CFA of nursing students’ Growth Mindset Scale.

Index	Acceptable value	Test results data
Absolute fit index		
χ^2^/df	< 3	1.423
RMSEA	< 0.08	0.045
Incremental fit index		
IFI	> 0.90	0.970
CFI	> 0.90	0.969
TFI	> 0.90	0.965
Parsimony fit index		
PGFI	> 0.05	0.710
PNFI	> 0.05	0.788
PCFI	> 0.05	0.845

### 4.5 Criterion-related validity

The correlations between the dimensions and total scores of Nursing Students’ Growth Mindset Scale and the ITIS ranged from 0.461 to 0.785 (*p* < 0.05), indicating good criterion-related validity (38).

### 4.6 Reliability testing

The Cronbach’s alpha of the NSGMS was 0.879, and the Cronbach’s alpha for 4 dimensions ranged from 0.766 to 0.901, revealing good internal consistency (41) (Table 4).

**Table 4:** Subdimensions, items, and Cronbach’ s alpha values of the scale.

Subscales	Number of items	Items	Score range	Cronbach’s alpha
Perception of talent and intelligence	5	1, 2, 3, 4, 5	1–5	0.901
Attitude toward challenges	6	6, 7, 8, 9, 10, 11	1–5	0.920
Attitude toward setbacks	5	12, 13, 14, 15, 16	1–5	0.866
Attitude toward others’ influences	5	17, 18, 19, 20, 21	1–5	0.766
Total	21	1–21	1–5	0.879

### 4.7 Stability testing

In this study, the correlation coefficient for the 2-week interval test–retest reliability was 0.901. The correlation coefficients of the subscales were as follows: 0.859 for perception of talent and intelligence, 0.905 for attitude toward challenges, 0.884 for attitude toward setbacks, and 0.909 for attitude toward the influence of others, indicating good stability of the scale (42) (Table 5).

**Table 5:** Test–retest reliability of the nursing students’ Growth Mindset Scale (*n* = 30).

Domains	Pearson
Attitude toward challenges	0.859
Perception of talent and intelligence	0.905
Attitude toward setbacks	0.884
Attitude toward others’ influences	0.909
Total	0.901

## 5 Discussion

In this study, by performing a comprehensive literature review and qualitative interviews and based on the growth mindset theory, the Nursing Students’ Growth Mindset Scale was developed. The scale addresses a gap in existing assessment tools, which are limited in their ability to capture the specific characteristics and learning contexts of nursing students. By integrating relevant theoretical frameworks and the actual experiences of nursing students, the scale was designed to reflect core features of growth mindset, while being tailored to the unique educational and clinical environment of nursing students. This design allows the scale to comprehensively assess nursing students’ growth mindset in a way that is both theoretically grounded and practically applicable.

The content validity of NSGMS was evaluated by 15 experts from 2 different fields: nursing education and nursing psychology. After a 2-round Delphi, the content validity of the draft questionnaire was ensured (45). A pilot implementation and subsequent item analysis further refined item wording and discrimination. The retained items effectively reflect nursing students’ growth mindset in domains such as beliefs about ability, responses to challenges, coping with setbacks, and susceptibility to others’ influence. For example, items such as “I believe that with my learning abilities, I can achieve certain success in the field of nursing.” and “I am willing to explore new challenges in the process of learning nursing skills.” illustrate how the scale captures learning situations and psychological demands specific to the nursing profession.

Subsequently, the EFA extracted four factors, namely perception of talent and intelligence, attitude toward challenges, attitude toward setbacks, and attitude toward others’ influence, with a cumulative variance contribution rate of 67%, confirming the rationality of the scale’s structure (46). These dimensions reflect the multifaceted connotations of nursing students’ growth mindset, such as beliefs in coping with clinical challenges, self-improvement in professional skills, responses to mistakes or negative feedback, and behavioral reactions under the influence of peers or evaluators. The CFA further indicated that the model had a good overall fit, demonstrating that the scale can effectively measure the multidimensional structure of nursing students’ growth mindset. In addition, the internal consistency and test–retest reliability of each dimension met psychometric standards, confirming its stability and reliability (47). Together, these findings not only support the scientific validity of the scale but also provide a basis for its application in nursing student training, screening, and clinical teaching practice.

During scale development and validation, two items, “My classmates are better than me, and I feel like a failure” and “Teachers/patients/classmates criticize me; I will be afraid to contact them again,” were removed because their correlation coefficient was below 0.4, which indicate the content of the items may not capture the key point of growth mindset. Another item, “The excellence of my classmates has nothing to do with me, and I will not change for it” was also removed because the factor loadings were below 0.5. This can be put down to the fact that students generally believed that they were influenced by others’ success instead of being indifferent, which is consistent with the growth mindset theory. Furthermore, in the EFA, the items in the “attitude toward others’ success” and “attitude toward others’ evaluation” dimensions were clustered in a dimension which was not consistent with the draft scale. There is a partial intersection between the two dimensions, which is mainly related to nursing students being affected by others. Therefore, they were combined into one dimension and labeled “attitude toward others’ influences.”

Compared with general instruments such as ITIS-3 and ITIS-6 (19, 24, 40), the NSGMS places greater emphasis on nursing student characteristics and better aligns with their practical circumstances. The scale identifies specific factors influencing nursing students’ growth mindset and complements general scales by offering more actionable information for nurse educators. Compared with other Growth Mindset Scales developed for nursing students (48), the NSGMS in this study differs in both the number and content of its dimensions, and its styles of expression also differ. The core beliefs and attitudes underlying growth mindset and contextual features unique to nursing students’ learning and clinical practice are taken into account in the development of NSGMS. First, as the essence of growth mindset theory, the perception of talent and intelligence was reflected in items 1–5. Besides universal contents such as the definition of talent and intelligence, the cognition of intellectual growth also focused on nursing students’ views on the role of endeavor and talent in nursing professional learning. Second, growth mindset will prompt nursing students to engage in positive behaviors in response to challenges and novel situations encountered during professional learning or extracurricular activities. The relevant content for this was incorporated in items 6–11. Moreover, the particular characteristics of nursing professionals’ theoretical learning and clinical practice were embodied in the scale, such as large quantities of mnemonic contents and the necessity of lifelong learning and looking death in the face. Third, previous research demonstrated that one’s attitude toward setbacks is crucial for an individual’s growth mindset, which the existing assessment tools lack. The measurement of nursing students’ emotions and behavior after facing setbacks in the process of nursing professional learning was incorporated into items 12–14 and items 15 and 16, respectively. Finally, nursing students’ growth mindset was reflected in their attitudes toward the influence of others, which included nursing students’ actions taken in response to and views on others’ success and the impact of others’ evaluation on them. The literature review and semi-structured interviews indicated that these individuals included teachers, classmates, and patients. Accordingly, the relevant content was embodied by items 17–21.

After continuous refinement and elaboration of its dimensions and items, the scale can be used to assist nursing educators in identifying students with lower levels of growth mindset and provide targeted support to enhance motivation, resilience, and coping skills. The results can be applied to design interventions that encourage positive attitudes toward challenges and strengthen clinical adaptation. Moreover, the scale offers a useful tool for monitoring students’ psychological well-being and academic development, supporting evidence-based teaching strategies and personalized educational plans.

However, this study also has several limitations. First, the sample’s demographic profile predominantly female and concentrated in the early years of professional training, may limit the generalizability of our findings across genders and career stages. Secondly, the sample size and regional scope were limited, which may render the findings insufficient to establish definitive cut-off values. Future studies with larger and more diverse samples are needed to identify appropriate ranges for nursing students’ growth mindset levels.

## 6 Conclusion

In this study, the NSGMS was developed and validated to assess growth-mindset in nursing students. This work resulted in a 21 items scale distributed into four dimensions, which showed good psychometric properties. The scale yields both a total score and subscale scores, offering a nursing specific complement to general growth-mindset measures. This scale may prove useful to better understand the role of nursing students’ growth mindset in academic performance, clinical practice adaptation, and psychological well-being. It can also be useful for descriptive assessment, screening, and to guide targeted educational interventions.

## Data Availability

The original contributions presented in the study are included in the article/supplementary material, further inquiries can be directed to the corresponding author/s.
